# Targeted Cancer Therapy: What’s New in the Field of Neuroendocrine Neoplasms?

**DOI:** 10.3390/cancers13071701

**Published:** 2021-04-03

**Authors:** Anna La Salvia, Paula Espinosa-Olarte, Maria Del Carmen Riesco-Martinez, Beatriz Anton-Pascual, Rocío Garcia-Carbonero

**Affiliations:** Oncology Department, Hospital Universitario 12 de Octubre, UCM, 28041 Madrid, Spain; alasalvi@ucm.es (A.L.S.); paula.espinosa@salud.madrid.org (P.E.-O.); criesco@salud.madrid.org (M.D.C.R.-M.); beatriz.anton@salud.madrid.org (B.A.-P.)

**Keywords:** neuroendocrine tumors, targeted therapy, personalized treatment, novel agents

## Abstract

**Simple Summary:**

Neuroendocrine neoplasms are highly heterogeneous tumors in terms of primary origin, molecular landscape, clinical presentation and behavior. To date, several drugs have been approved and many ongoing trials are testing new agents or new combinations. In this work we aim to provide a comprehensive review of approved agents and promising novel drugs in clinical development for the treatment of neuroendocrine neoplasms. Our manuscript could be a useful review and guidance for neuroendocrine neoplasms-dedicated clinicians.

**Abstract:**

Neuroendocrine tumors (NETs) are a heterogeneous family of neoplasms of increasing incidence and high prevalence due to their relatively indolent nature. Their wide anatomic distribution and their characteristic ability to secrete hormonally active substances pose unique challenges for clinical management. They are also characterized by the common expression of somatostatin receptors, a target that has been extremely useful for diagnosis and treatment (i.e., somatostatin analogues (SSAs) and peptide-receptor radionuclide therapy (PRRT)). Chemotherapy is of limited use for NETs of non-pancreatic origin, and the only approved targeted agents for advanced progressive NETs are sunitinib for those of pancreatic origin, and everolimus for lung, gastrointestinal and pancreatic primaries. Despite recent therapeutic achievements, thus, systemic treatment options remain limited. In this review we will discuss the state-of-the-art targeted therapies in the field of NETs, and also future perspectives of novel therapeutic drugs or strategies in clinical development, including recently presented results from randomized trials of yet unapproved antiangiogenic agents (i.e., pazopanib, surufatinib and axitinib), PRRT including both approved radiopharmaceuticals (177Lu-Oxodotreotide) and others in development (177Lu-Edotreotide, 177Lu-Satoreotide Tetraxetan), immunotherapy and other innovative targeted strategies (antibody-drug conjugates, bites,…) that shall soon improve the landscape of personalized treatment options in NET patients.

## 1. Introduction

Neuroendocrine neoplasms (NENs) are a heterogeneous family of tumors that originate from the diffuse neuroendocrine system. Although traditionally considered rare tumors, their incidence has substantially increased over the last decades, reaching 6.98 new cases/100,000 inhabitants/year [1,2,3], and their prevalence is high due to their relatively indolent nature [1]. NENs are classified according to the World Health Organization (WHO) classification, based on tumor differentiation and proliferation rate. Approximately 80% of all NENs are well-differentiated tumors (NETs), the majority of which present a low proliferation rate (mitotic count < 20 HPFs and/or Ki-67 index < 20%) and are classified as G1 or G2 NETs. A small subset of NETs may however have a proliferation index greater than 20% (G3 NETs), and this entity has been recently recognized in the 5th edition of the WHO Classification of Tumors of the Digestive System published in August 2019 [4]. This group biologically resembles low grade tumors, although it is associated with a more aggressive clinical behavior. NETs can be classified as functioning (~20%) or non-functioning depending on their capacity to produce hormones (i.e., insulin, glucagon, gastrin, vasoactive intestinal peptide or somatostatin), peptides and neurotransmitters (i.e., serotonin). Excessive production of these hormones or peptides may be associated with specific clinical syndromes and is a distinctive feature of NETs. Poorly differentiated neuroendocrine carcinomas (NECs) substantially differ from NETs in terms of biologic aggressiveness, response to treatments and prognosis [5,6]. NECs have always a high proliferative index (Ki-67 > 20% or G3), less frequently express somatostatin receptors, rarely produce a hormonal syndrome and have a very poor overall survival. The majority of NENs are originated in the gastroenteropancreatic (GEP) or bronchopulmonary tracts, although they may develop in any organ. This high complexity and clinical heterogeneity, including their wide anatomic distribution and their characteristic ability to secrete hormonally active substances, pose unique challenges for clinical management. The treatment strategy widely varies according to a number of factors, such as primary tumor site, histological features (tumor differentiation, proliferation rate, expression of somatostatin receptors (SSTR), clinical presentation (tumor- or hormone-related symptoms, performance status, comorbidities) and disease stage. A multidisciplinary tumor board evaluation in NET-specialized centers is thus highly encouraged in order to define an optimal personalized strategy.

Surgery is the only curative approach for NENs. However, surgical excision is not always possible as 50–60% of patients present metastatic disease at diagnosis [7,8]. In patients with locally advanced inoperable or metastatic NENs, treatment goals include tumor growth control and symptom relief. In this context, systemic therapy is the standard of care, although cytoreductive surgery and regional approaches may also be considered. Local cytoreductive/ablative therapies are most commonly used in patients with liver-dominant disease, and include radiofrequency ablation, bland embolization or chemo- or radio-embolization with Yttrium-90-labeled microspheres. Systemic treatment options have progressively increased over the last decades, and comprise biotherapy, targeted agents, chemotherapy regimens and radiopharmaceuticals. The characteristic and common expression of SSTR on NET cells surface has been extremely useful for diagnostic imaging with111In-Octreotide scintigraphy, or with the more sensitive 68Ga-based PET/CT and also for treatment (i.e., somatostatin analogues (SSAs) and peptide-receptor radionuclide therapy (PRRT)). Biotherapy with SSAs has traditionally been considered the mainstay of systemic therapy for low grade NETs, given their efficacy to control hormonal production excess and because of their proven antiproliferative activity [9,10]. An increasing body of evidence has also demonstrated the effectiveness and safety of PRRT for SSTR-positive NETs [11,12,13]. Beyond SSTR-targeted therapy, only two other targeted agents have been approved to date for NETs: the antiangiogenic sunitinib for those of pancreatic origin [14], and everolimus for lung, gastroenteropancreatic (GEP) NETs or NETs of unknown origin [15,16]. Chemotherapy is the standard of care for aggressive, poorly differentiated NECs, but its use in NETs is limited to those of pancreatic origin or rapidly progressive extra-pancreatic NETs who have failed other more effective therapeutic options [17,18].

In summary, despite recent therapeutic achievements, systemic treatment options remain limited and a consensus on the optimal treatment sequence in patients with advanced disease is still lacking.

In this manuscript we will review the state-of-the-art targeted therapies in the field of NENs and discuss future perspectives of novel therapeutic drugs or strategies in clinical development, including recently presented results from randomized trials of yet unapproved antiangiogenic agents (i.e., pazopanib, surufatinib and axitinib), PRRT including both approved radiopharmaceuticals (177Lu-Oxodotreotide) and others in development (177Lu-Edotreotide, Satoreotide Tetraxetan), immunotherapy and other innovative targeted strategies (antibody-drug conjugates) that shall soon improve the landscape of personalized treatment options in NET patients.

## 2. mTOR Pathway: Relevance in NETs

### 2.1. Rationale for Targeting the mTOR Pathway

The mammalian target of rapamycin (mTOR) is an intracellular highly conserved serine/threonine kinase that acts as the catalytic subunit of two structurally and functionally distinct multiprotein complexes, mTOR complex 1 (mTORC1) and mTORC2, that play key roles in regulating physiological anabolic and catabolic processes in response to external cues. mTOR is the most important downstream component of the phosphatidylinositol-3-kinase (PI3K)/AKT signaling pathway. This network regulates essential cellular functions such as cellular proliferation, metabolism and apoptosis [19,20,21,22]. The PI3K/AKT/mTOR signaling pathway plays a crucial role in controlling cancer cell-cycle and growth [23]. Mutations of mTOR signaling components provide cancer cells with a selective growth advantage with respect to normal cells [24,25,26]. This pathway is dysregulated in a broad variety of human tumors, including NETs [27,28,29,30,31,32,33,34,35,36,37,38,39,40,41,42]. Sequencing studies of pancreatic (P-NET) and small intestinal (SI-NET) NETs showed that 14 and 33% of cases, respectively, harbored mutations in at least one gene encoding for mTOR pathway components [43,44,45,46,47,48]. High expression of mTOR or its activated downstream targets p-RPS6KB1, p-RPS6 or p-EIF4EBP1 was associated with higher tumor proliferative capacity, a more aggressive clinical behavior and a shorter survival [49,50,51]. Based on this biological rationale, the anti-proliferative effect of mTOR pathway inhibition was identified as a promising therapeutic strategy in cancer and in NETs [52,53]. Rapamycin was discovered as a potent antifungal agent, but it also exhibited immunosuppressive properties, which subsequently led to its clinical development to prevent rejection of solid-organ transplantation. Rapamycin binds to the intracellular receptor, FKBP12, thus interacting with mTORC1 and preventing the downstream pathway’s activation [54]. However, rapamycin is an oral drug with low bioavailability. In addition to rapamycin, several rapamycin analogs (“rapalogues”) have been developed such as CCI779 (temsirolimus) and RAD001 (everolimus), among others. Rapalogs have a similar mechanism of action, but improved pharmacodynamic and pharmacokinetic characteristics. These compounds have demonstrated antiproliferative activity in vitro and in vivo, both in NET cell lines (BON-1) and preclinical models [55,56].

### 2.2. mTOR Inhibitors: Everolimus and Beyond 

The efficacy and safety of mTOR inhibitors has been demonstrated in different tumors, including lymphomas, breast and renal cell carcinomas (RCC) [57,58]. A robust antitumor activity of everolimus has also been consistently demonstrated in the phase II/III RADIANT trials across a broad spectrum of NETs including those arising from the pancreas, lung and gastrointestinaltract [15,16,59,60,61]. The study designs and results of these trials are summarized in Table 1. The most frequent adverse events (AEs) observed with everolimus were generally of grade 1 or 2, and included stomatitis, diarrhea, fatigue, infections, rash and peripheral oedema. Most everolimus-related AEs were manageable through dose interruption and/or modification without altering the duration of treatment. Based on these results, the Food and Drug Administration (FDA) and the European Medicines Agency (EMA) approved everolimus for the treatment of unresectable or metastatic, non-functional G1-G2 NETs of GI or lung origin or NETs of pancreatic origin in adults with progressive disease. Notably, clinical trials specifically exploring everolimus in G3 NETs are currently ongoing (ClinicalTrials.gov Identifier: NCT02113800, NCT02248012).

### 2.3. Everolimus-Based Combinations 

Mechanisms of innate and acquired resistance to mTOR inhibition include the activation of several compensatory signaling pathways, upstream activation of PI3K/AKT signaling, the occurrence of FKBP12 or mTOR mutations, epigenetic alterations, compensatory metabolism rewiring or the stimulation of autophagy [66,67]. To try to overcome resistance, different everolimus-based combinations have been explored, particularly with SSAs and antiangiogenic agents [62,63,65,68,69,70,71,72,73]. Randomized trials do not suggest a clear benefit in terms of efficacy for these combinations (Table 1*)*, whereas some safety concerns were raised for some of these combinations.

Currently, several trials are assessing the combination of everolimus with other treatment strategies such as PRRT, chemotherapy or other targeted agents, such as the phase I-II study testing the combination with Lu-177-DOTATATE therapy in GEP or lung NETs (ClinicalTrials.gov Identifier: NCT03629847), the phase II trial evaluating the association with LEE011 (Ribociclib) in advanced foregut NETs (ClinicalTrials.gov Identifier: NCT03070301) and the phase I-II study testing the combination with temozolomide in advanced P-NETs (ClinicalTrials.gov Identifier: NCT00576680).

### 2.4. Other mTOR inhibitors: Temsirolimus and Sapanisertib

Temsirolimus is an intravenous mTOR inhibitor that has been investigated in few phase II clinical trials enrolling NET patients. As a single agent it showed limited activity (objective response rate (ORR) of 5.6%) [74], but results in combination with bevacizumab were encouraging [75], with an ORR of 41%, a median progression-free survival (PFS) of 13.2 and a median overall survival (OS) of 34 months. The safety profile was manageable. Randomized trials assessing the addition of bevacizumab to everolimus or to octreotide depot, however, failed to demonstrate a clear benefit [65], and the temsirolimus–bevacizumab combination was not further developed in this setting.

Dactolisib (BEZ235) is an oral dual PI3K/mTOR inhibitor that selectively inhibits class I PI3K (p110α, -β, -δ and -γ), mTORC1 and mTORC2 by reversibly binding to the ATP-binding sites of kinases and inhibiting their catalytic activity. Notwithstanding, it did not demonstrate greater efficacy as compared to mTORC1 inhibition alone by everolimus in patients with P-NETs and was significantly more toxic [64]. Clinical development of this agent was halted.

Sapanisertib (INK128) is a second-generation ATP-competitive mTOR kinase inhibitor that potently suppresses both mTORC1 and mTORC2 [76], overcoming resistance to everolimus induced by phosphorylation of 4EBP1 and AKT [77]. Preclinical studies have shown sapasertib has antitumor activity in everolimus-resistant P-NET patient-derived xenograft models [78]. Clinical trials are currently evaluating Sapanisertib efficacy and safety in different clinical settings, including rapalog-resistant advanced P-NETs (ClinicalTrials.gov Identifier: NCT02893930).

## 3. Role of Angiogenesis in NETs

### 3.1. Rationale for Targeting Angiogenesis 

Angiogenesis is one of the hallmarks of cancer as it plays a key role in providing oxygen and nutrients for tumor cell growth and progression [79]. Targeting angiogenesis has been successfully explored as a therapeutic strategy in a wide spectrum of solid tumors, including NETs. Angiogenesis is a highly controlled process tightly regulated by a complex equilibrium of pro- and anti-angiogenic factors secreted by tumor cells and by cells of the tumor microenvironment (pericytes, mesenchymal, endothelial or immune cells). Among these, the vascular endothelial growth factor (VEGF) is particularly relevant. VEGF stimulates both the proliferation and migration of endothelial cells, enhances vascular permeability, vasodilatation and the recruitment of inflammatory cells and is essential for revascularization during tumor formation [80]. In mammals, five members of the VEGF family have been identified, VEGF-A being the most potent stimulator of angiogenesis [81]. Downstream signaling of VEGF in tumor cells is mediated by a family of receptor tyrosine kinases, including VEGFR-1,2,3 [82]. The production of VEGF is regulated by local oxygen availability through the hypoxia-inducible factor-1 (HIF-1) in a dynamic process that results in the transcription of several genes involved in proliferation, angiogenesis, survival and apoptosis [83,84].

Multiple approaches have been developed to target angiogenesis over the last decades and several antiangiogenic drugs have been approved as oncological therapies [85,86]. Notably, the rich vascularization is a typical feature of well-differentiated NETs. This characteristic is associated with the overexpression of both VEGF ligands and their receptors [87] in 60–84% of cases [88]. NETs also show a high expression of platelet-derived growth factor receptors (PDGFRs) α and β, as well as stem-cell factor receptors (c-kit). These factors have also been involved in NET development and progression. Several lines of evidence show that the dense vascular network associated with low-grade NETs are more likely to be a marker of differentiation than a marker of aggressiveness, as opposed to what is observed in other epithelial tumors. This phenomenon represents the so-called ’neuroendocrine paradox’, meaning that vascularization is inversely related to the aggressiveness of the disease. However, other studies observed that VEGF over-expression was correlated with a worse clinical outcome in patients with well-differentiated NETs [89].

Based on this strong rationale, an increasing number of clinical trials evaluating the activity of different agents with antiangiogenic properties have been conducted in advanced NETs. The results of the most relevant phase II-III trials are summarized in Table 2.

### 3.2. Angiogenesis Inhibitors Assessed in Phase III Randomized Trials

#### 3.2.1. Sunitinib

Sunitinib is a multi-tyrosine kinase inhibitor (TKI) that targets VEGFR, PDGFR, stem-cell factor receptor, glial cell line-derived neurotrophic factor receptor (GDNF) and FMS-like tyrosine kinase-3 [96]. To date, this compound has been approved for the treatment of patients with advanced gastrointestinal stromal tumors (GIST), RCC and P-NETs [14,97,98].

The EMA in 2010 and the FDA in 2011 approved sunitinib for advanced progressive P-NETs based on the results of an international investigator-initiated randomized doble-blind placebo-controlled phase III study that demonstrated a significant improvement in PFS (11.4 vs. 5.5 months, HR 0.42, *p* < 0.001) and OS (HR 0.41, *p* = 0.02) for sunitinib-treated patients as compared to those treated with placebo [14]. Upon study closure, 69% of placebo-treated patients crossed over to sunitinib, which likely diluted the early impact observed on OS. With five additional years of follow-up, median OS was 38.6 months for sunitinib and 29.1 months for placebo (HR: 0.73; *p* = 0.094) [99]. The most frequent adverse events in the sunitinib group were diarrhea, nausea, vomiting, asthenia and fatigue. An updated safety analysis of sunitinib- and placebo-treated patients from this study that continued to receive sunitinib in two open-label extension studies confirmed sunitinib was well-tolerated in the long term and the safety profile was consistent with that reported in the original pivotal Phase III study [100].

#### 3.2.2. Surufatinib

Surufatinib is an orally bioavailable, small molecule inhibitor that targets VEGFR-1,2,3, fibroblast growth factor receptor type 1 (FGFR1) and colony-stimulating factor-1 receptor (CSF-1R) [101]. Activation of the FGFR pathway contributes to both intrinsic and acquired resistance to the VEGF blockade. Both FGF and CSF-1 signaling are involved in immune evasion through the recruitment and maintenance of myeloid-derived suppressor cells and tumor-associated macrophages to the tumor microenvironment. The inhibition of VEGFRs and FGFR1 may lead to a more potent angiogenesis blockade, that, together with the simultaneous depletion of peritumoral immunosuppressive cells, might enhance antitumor activity. A multicenter, single-arm, open-label phase Ib/II trial, including 42 P-NETs and 39 extra-pancreatic NETs, showed encouraging antitumor activity and manageable toxicity [102]. Based on these results, two phase III studies were undertaken. First, the SANET-ep study (NCT02588170) included 198 patients with extra-pancreatic NETs that were randomized 2:1 to receive surufatinib 300 mg daily (*n* = 129) or placebo (*n* = 69) [91]. The most common primary tumor site was the rectum (27% of patients), followed by the lung, thymus, stomach and small bowel (8%). The median PFS per investigator assessment was 9.2 months for patients treated with surufatinib, as compared to 3.8 months for patients in the placebo group (HR 0.33; *p* < 0.0001). The study was terminated early as it met the predefined criteria for early discontinuation at the interim analysis. The efficacy of surufatinib was seen across all subgroups and further supported by significant improvements in secondary efficacy endpoints including ORR (10% vs. 0%, *p* = 0.0051), DCR and duration of response. Efficacy was confirmed by the Blinded Independent Image Review Committee (“BIIRC”) assessment, although the magnitude of the effect on PFS seemed somewhat lower (HR 0.66, *p* = 0.037). OS data was not mature, as only 21% of patients treated with surufatinib and 14% of those treated with placebo had died at the time of interim analysis. Surufatinib was generally well-tolerated. The most common treatment-related grade >3 AEs were hypertension (36 vs. 13%) and proteinuria (19 vs. 0%). The second pivotal phase III clinical trial was the SANET-p study, that randomized (2:1) 264 patients with advanced P-NETs to receive surufatinib or placebo [90].

As the SANET-ep trial, this study was terminated early as it met the pre-specified early stopping criteria at interim analysis. The median investigator-assessed PFS was 10.9 versus 3.7 months for surufatinib- and placebo-treated patients, respectively (HR 0.49, *p* = 0.0011). ORR was also significantly greater in patients treated with surufatinib (19%) compared to patients treated with placebo (2%) (*p* = 0.002). Overall responses and PFS by BIIRC assessment were similar to those reported by the investigators. The most common grade 3 or worse treatment-related AEs were hypertension (38 vs. 7%), proteinuria (10 vs. 2%) and hypertriglyceridaemia (7% vs. none). Treatment-related serious AEs were reported in 22 (surufatinib) vs. 7% (placebo) of patients. QoL assessments were similar in both study arms except for diarrhoea domain scores, that were worse for surufatinib-treated patients. Based on these pivotal studies, that were fully conducted in Chinese populations, the National Medical Products Administration (NMPA) very recently approved surufatinib for the treatment of advanced non-pancreatic NETs and it will likely be approved soon for pancreatic primaries. A phase I US trial showed similar surufatinib pharmacokinetics in Caucasian patients, and further supportive studies are planned to be conducted in western countries to pursue FDA and EMA approval.

#### 3.2.3. Axitinib

Axitinib is a potent second-generation TKI that selectively inhibits VEGFR-1,2,3 and has demonstrated activity against other vascular-dependent solid tumors such as RCC [103,104]. An open-label, phase II trial that assessed axitinib in 30 patients with extra-pancreatic NETs reported a median PFS of 26.7 months and a median OS of 45.3 months. The best objective response in this trial was partial response (PR) in 1/30 (3%) and stable disease (SD) in 21/30 patients (70%). Hypertension was developed in a high proportion of patients (90%), being of grade 3/4 in 19 patients (63%) and leading to treatment discontinuation in six (20%) [105]. A phase II/III randomized double-blind study, the AXINET trial, was conducted by the Spanish Cooperative Group of Neuroendocrine and Endocrine Tumors (GETNE) to evaluate the efficacy of axitinib in combination with octreotide long-acting repeatable (LAR) versus placebo and octreotide LAR, in 256 patients with advanced G1-G2 NETs of non-pancreatic origin (NCT01744249) [92].

The ORR was significantly greater in the axitinib arm (17.5 vs. 3.8% for axitinib- and placebo -treated patients, respectively, *p* = 0.0004). The median PFS per investigator assessment was 17.2 months for the axitinib–octreotide arm versus 12.3 months for the placebo–octreotide arm, but this difference did not reach statistical significance (HR 0.816, *p* = 0.169). Hypertension was reported in 50% of patients (21% of grade 3–4), Other grade 3–4 AEs more commonly observed in axitinib-treated patients were diarrhea (14 vs. 2%) and fatigue (9 vs. 3%). Independent blinded radiological assessment of PFS is currently ongoing and expected to be reported in the very near future.

#### 3.2.4. Cabozantinib

Cabozantinib is an oral small molecule multikinase inhibitor that targets, among others, VEGFR, MET and RET. This drug was assessed in a two-cohort phase II trial that included 20 P-NETs and 41 extra-pancreatic NETs (NCT03375320) [106]. Treatment with cabozantinib was associated with objective tumor responses (ORR of 15% in both cohorts) and encouraging PFS (22 and 31 months in patients with advanced NETs of pancreatic and extra-pancreatic origin). Grade 3–4 toxicity included hypertension (13%), hypophosphatemia (11%), diarrhea (10%), lymphopenia (7%), thrombocytopenia (5%), fatigue (5%) and increased lipase or amylase (8%). A phase III double-blind randomized trial (CABINET) is currently testing cabozantinib versus placebo in advanced NETs pretreated with at least one FDA-approved drug (except somatostatin analogues) (NCT03375320). Results shall be available in the upcoming years and are awaited with great interest.

#### 3.2.5. Bevacizumab

Bevacizumab is a humanized anti-VEGF monoclonal antibody that has shown relevant anti-tumor activity in a variety of solid neoplasms. A small randomized phase II trial suggested this drug was also active in GEP-NETs [107]. In this study, 44 patients on stable doses of somatostatin analogues were randomly assigned to receive single-agent bevacizumab or PEG interferon alfa-2b (IFN- α-2b) for up to 18 weeks. Thereafter, or at disease progression, whichever occurred first, patients were allowed to receive both drugs in combination. Patients treated with bevacizumab achieved a higher response rate (18 vs. 0%) and PFS rate at 18 weeks (95 vs. 68%), and a significant decrease in tumor blood flow assessed by functional CT scans that was not observed in INF-treated patients. Based on these encouraging results, a large phase III trial (SWOG S0518) was designed to compare octreotide LAR and bevacizumab or IFN-α-2b in 427 patients with advanced G1-2 carcinoids [94]. ORR were significantly greater for bevacizumab-treated patients, although modest in both study arms (12 vs. 4%, *p* = 0.008). The median PFS by central review was not significantly different among study arms (16.6 vs. 15.4 months in the bevacizumab and IFN arms, respectively, HR 0.93; *p* = 0.55). The time to treatment failure was longer with bevacizumab (HR 0.72; *p* = 0.003). This may have been due to differences in the toxicity profiles of study drugs. Bevacizumab’s most common side effects were hypertension and proteinuria, easily manageable, whereas over 25% of interferon-treated patients experienced grade 3–4 fatigue, which likely justified the higher proportion of patients that withdrew consent in this study arm. The authors concluded that both agents had similar antitumor activity in patients with advanced NETs, although it is unlikely that any of these agents will ever be approved in this context by regulatory agencies.

The results of single-arm studies exploring the combination of bevacizumab with mTOR inhibitors suggested a synergistic effect (ORRs of 21% for bevacizumab and everolimus, and of 41% for bevacizumab and temsirolimus) [72,108]. The randomized phase 2 CALGB 80701 trial confirmed the ORR was significantly greater for the everolimus–bevacizumab combination versus single-agent everolimus (31 vs. 12%, *p* = 0.005), although this only translated into a modest increase in PFS (16.7 vs. 14.0 months, *p* = 0.12) [109].

### 3.3. Angiogenesis Inhibitors in Earlier Stages of Clinical Development

#### 3.3.1. Sorafenib

Sorafenib is an orally administered TKI that targets the RAF/MEK signaling pathway as well as VEGFR, PDGFRs, FLT3 and c-KIT. It was approved for the treatment of advanced hepatocellular carcinoma and RCC [110,111]. Sorafenib was also tested in NETs. A Phase II trial assessed sorafenib in 93 patients with advanced P-NETs and carcinoid tumors [112]. An ORR of 10% was observed in both groups. PFS rates at 6 months were 40% for carcinoid tumors and 61% for P-NETs. Grade 3–4 toxicity occurred in 43% of patients, with skin toxicity (20%), diarrhea (7%) and fatigue (9%) being the most commonly encountered. Further clinical development in prospective randomized trials was not pursued for this drug in NETs, although the antitumor activity of this agent did not seem to be substantially different from that reported with other targeted agents in this context. Sorafenib was also explored in combination with other drugs such as everolimus [73] or bevacizumab [113], with no clear benefit in terms of efficacy and significantly increased toxicity.

#### 3.3.2. Pazopanib

Pazopanib is another TKI inhibiting VEGFRs, PDGFRs and c-Kit [114] that is approved for the treatment of RCC and soft tissue sarcoma [115,116]. Single-arm phase II studies have explored pazopanib in NETs of different primary sites, with ORRs of 10–22% and a median PFS of 9–14 months [117,118]. Interestingly, pazopanib also showed activity in patients pre-treated with other targeted therapies and in G3 NETs (ORR 23%) [119]. The results of a multicenter, randomized, double-blind phase II trial (A021202) comparing pazopanib to placebo in advanced extra-pancreatic NETs were presented at the ASCO Annual Meeting in 2019 [93]. This study enrolled 171 patients, 66% of them with small bowel primary tumors and 87% receiving concurrent SSA. The median PFS was 11.6 vs. 8.5 months in the pazopanib and placebo arms, respectively (HR = 0.53, *p* = 0.0005), which crossed the pre-specified protocol efficacy boundary. Some degree of tumor shrinkage was achieved in 55 and 31% of pazopanib- and placebo-treated patients, respectively, although objective responses were only documented in two patients (2%) of the pazopanib arm. The OS was not significantly different among study arms (median of 41 and 42 months, HR = 1.13, *p* = 0.70). Treatment-related grade 3–4 AEs occurred in 61% of patients treated with pazopanib vs. 21% of patients in the placebo arm. The most common severe side effects of pazopanib were hypertension (27%) and hypertransaminasemia (9%). QoL analysis documented that patients treated with pazopanib experienced more symptoms (diarrhea, appetite loss, dyspnea, fatigue, nausea and vomiting), but the overall QoL was similar among study arms.

#### 3.3.3. Lenvatinib

Lenvatinib is another TKI that targets VEGFR1-3, FGFR1-4, PDGFRα, c-Kit and RET [120]. This compound was recently approved for the treatment of radioiodine-refractory differentiated thyroid cancer and has been also tested in NETs. In the phase II TALENT clinical trial (GETNE1509), lenvatinib was assessed in two cohorts; the first included 55 P-NETs, and the second, 56 gastrointestinal NETs (GI-NETs) [121]. For P-NETs, the ORR by central radiology assessment was 40.4%, the highest ever reported for a TKI in this setting, with a median PFS of 15.5 months. For GI-NETs, the ORR was 16.3% and the median PFS 15.4 months. Lenvatinib was administered at a dose of 24 mg qd but dose reductions/interruptions were required in 88% of patients. The most frequent grade 3–4 AEs were hypertension (22%), fatigue (11%) and diarrhea (11%).

#### 3.3.4. Nintedanib

Nintedanib is a potent oral inhibitor of VEGFR, PDGFR and FGFR that is approved for the treatment of idiopathic pulmonary fibrosis. An open label phase 2 study was conducted in 32 patients with extra-pancreatic NETs that were treated with nintedanib and octreotide LAR [122]. The best response was stable disease in 26 patients (81%) and one patient achieved a partial response. The median PFS and OS was 11.0 and 32.7 months, respectively. Nintedanib was well-tolerated and delayed deterioration in quality of life. Increased serotonin levels were correlated with markers of impaired antitumor immunity.

#### 3.3.5. Aflibercept

Aflibercept is a recombinant fusion protein that consists of portions of the extracellular VEGFR-1 and -2 domains fused to the Fc portion of human immunoglobulin G1 [123]. It binds to both sides of the VEGF dimer, forming a so-called VEGF-trap, and exhibits higher affinity for VEGF-A/B but binds to all VEGF isoforms (VEGF-A, B, C and placental growth factor). Preclinical studies suggested activity of this compound in NENs [124,125]. Recently, a phase II open-label study, enrolling 21 patients with advanced P-NETs, reported an ORR of 9.5%, a median PFS of 15 months and a median OS of 34 months [126]. The most frequent treatment-related AEs were hypertension (77% of patients), headache (68%), mucositis (45%), hoarseness (41%) and proteinuria (32%). Proteinuria led to treatment discontinuation in five patients and one patient died due to a GI hemorrhage.

## 4. Somatostatin Receptors and Other Unique Targets in NETs

### 4.1. Rationale for Targeting SST

The majority of NETs are characterized by the expression of somatostatin receptors (SSTRs) on the cell membrane, a unique feature of NETs that has been very useful for diagnosis and therapy. Five different, G-protein-coupled SSTR subtypes (SSTR 1–5) have been identified. Their natural ligand, somatostatin (SST), is a neuropeptide secreted in the GI tract and the brain that regulates multiple physiological functions, such as neurotransmission, GI motility, hormone secretion, cell proliferation and apoptosis and immune system modulation [127]. The clinical utility of native human somatostatin was limited by its short half-life, thus SSAs were developed with a prolonged plasma half-life that facilitated clinical use [128]. The results of randomized phase III trials with SSTR-targeted agents in NETs are summarized in Table 3.

### 4.2. Somatostatin Analogues (Octreotide, Lanreotide, Pasireotide)

SSAs are synthetic octapeptides, with a longer half-life than native somatostatin 14 and 28, that enable clinical use. They have a similar STTR binding profile, with high SSTR2 and moderate SSTR5 affinity. SSAs are very effective drugs for hormonal syndrome control in functioning tumors [131], and also exert an antiproliferative effect by inducing cell cycle arrest and apoptosis, and through immunomodulatory effects and angiogenesis inhibition. Two randomized phase III trials demonstrated the antiproliferative effect of SSAs in the clinic. First, the PROMID study randomized 85 G1 advanced midgut NETs to receive octreotide LAR 30 mg every 4 weeks or placebo. A significant PFS improvement was reported for octreotide-treated patients (14.3 vs. 6 months, HR 0.34, *p* = 0.000072) [9]. Second, the CLARINET trial enrolled 204 patients with advanced non-functional GEP-NETs, with a Ki-67 index < 10% and a positive somatostatin-receptor scintigraphy [10]. PFS was significantly increased in patients treated with lanreotide as compared to placebo (median not reached vs. 18 months, HR = 0.47, *p* = 0.0002). Neither the PROMID nor the CLARINET studies demonstrated a benefit in terms of OS, although this endpoint is difficult to assess in the context of a very indolent disease, with a low rate of events, a high rate of crossover from the placebo group to SSA therapy and the potential confounding effect of subsequent lines of therapy upon disease progression.

Pasireotide is a second-generation SSA with greater binding affinity to SSTR1,2,3,5 currently approved for the treatment of Cushing’s syndrome and acromegaly, refractory to other somatostatin analogues. Due to its wider binding profile, pasireotide was expected to have greater antisecretory and antiproliferative activity than first-generation SSAs. Early studies demonstrated that it improved hormonal syndrome control in functioning NETs resistant to first-generation SSAs at conventional doses and also reported objective responses in some patients [132,133]. A phase 3 double-blind trial was then conducted in patients with digestive NETs with refractory carcinoid syndrome that were randomly assigned (1:1) to receive pasireotide LAR (60 mg) or octreotide LAR (40 mg) every 28 days [129]. The primary endpoint was symptom control based on the frequency of flushing episodes and bowel movements. The study was terminated early at the interim analysis for futility. Similar proportions of patients receiving pasireotide LAR (20.9%) or octreotide LAR (26.7%) achieved symptom control at 6 months (OR, 0.73; *p* = 0.53). Notably, a post hoc analysis observed a significantly longer PFS for pasireotide-treated patients than for patients treated with octreotide (11.8 vs. 6.8 months, HR 0.46, *p* = 0.045). However, the COOPERATE-2 trial failed to demonstrate any PFS benefit of adding pasireotide to everolimus versus everolimus alone in patients with pancreatic NETs, whereas the combination was more toxic (grade 3–4 hyperglycemia occurred in 37 versus 11% of patients) [62].

### 4.3. Radiopharmaceuticals Targeting SST

#### 4.3.1. Agonists (*β* and α Particle-Emitting Radionuclides) and Intra-Arterial PRRT

SSTR may also be targeted with radiolabeled SSAs such as 177Lu-DOTA-D-Phe-Tyr3-octreotate (177Lu-Oxodotreotide or 177Lu-DOTATATE) for peptide receptor radionuclide therapy (PRRT). Radiolabeled SSAs, upon binding to SSTRs on the NET cell surface, are internalized via endocytosis thereby causing selective DNA damage [134]. Patients with adequate kidney and bone marrow functions and a life expectancy greater than 3 months are suitable candidates for PRRT. In clinical practice, the approved indications are limited to G1-G2 well-differentiated metastatic NETs. NETs with a high and homogeneous SSTR expression, a low tumor burden and a slow growth rate are probably the optimal candidates for PRRT. The objectives of treatment are tumor growth control in patients with progressive disease, as well as symptomatic control in the context of hormone-secretory syndromes or tumor-related symptoms. Furthermore, recent and encouraging evidence is arising about the potential role for PRRT in the neoadjuvant setting. In this context, 177Lu-DOTATATE was reported to convert 15 out of 57 (26.3%) unresectable primary GEP NETs into resectable ones in a small non-controlled study [135]. Further prospective and randomized studies are needed to confirm these promising data.

Some contraindications to PRRT should also be noted. These include tumors with significant sites of SSTR-negative active disease, confirmed by 18F-FDG-PET if available, and patients with a poor general condition (Karnofsky performance status < 50%), insufficient bone marrow reserve (hemoglobin < 5 mmol/L (8 g/dL); platelet count < 75 × 10 ^9^ /L; white blood cell count < 2 × 10 ^9^ /L) or severe renal (creatinine clearance < 30 mL/min), liver (otal bilirubin > 3 × ULN; or both albumin < 25 g/L and prothrombin time increased > 1.5 × ULN) or cardiac (New York Heart Association grade III or IV; moderate to severe right heart valvular disease) impairment. Pregnancy and ongoing lactation are also contraindications for PRRT.

177Lu-DOTATATE is a medium-energy β-emitter with a maximum energy of 0.5 MeV, a maximum tissue penetration of 2 mm and a half-life of 6.7 days. Lutetium-177 also emits low-energy γ-rays, allowing scintigraphy and subsequent dosimetry with the same therapeutic compound, if needed. The shorter β-range of 177Lu compared to other radioisotopes such as yttrium (range of 12 mm) improves safety as it spares surrounding healthy tissue from radiation [136]. Its relatively long half-life also provides logistic advantages as it facilitates its supply to locations far from reactors. In 2017/2018, the EMA/FDA approved 177Lu-DOTATATE for use in SSTR-positive G1-2 GEP-NETs, based on results of the phase III NETTER-1 trial [13]. This study randomized (1:1) 229 patients with midgut NET, who presented with progressive disease on standard-dose octreotide LAR (20–30 mg every 4 weeks), to receive 177Lu-DOTATATE at a dose of 7.4 GBq every 8 weeks (four intravenous infusions) plus octreotide LAR 30 mg every 4 weeks, or high doses of octreotide LAR alone (60 mg every 4 weeks). Notably, the control arm of this study was an FDA-approved off-label use of high dose octreotide. PRRT demonstrated a pronounced positive effect on PFS compared to high dose SSAs (28 vs. 8.4 months, HR 0.21, *p* = 0.001), with a trend towards an improved overall survival (data still immature) and a favorable toxicity profile.

Combinations of Lu-177-labeled peptides with Y-90-labeled peptides or with other agents are being actively investigated and may prove to be of additional therapeutic benefit. The rationale of combining the two isotopes is based on their different emission profile. Y-90 emits beta particles with a high maximum energy higher than Lu-177 and longer maximum particle range in tissues (10 mm). It is hypothesized that ^90^Y may be more adequate to treat larger tumors while ^177^Lu, with a shorter beta particle range and a longer half-life, may be preferable for small tumors. The combination of both isotopes may therefore be considered for patients with tumors of various sizes and non-homogeneous receptor distribution. Initial data indicate that combination treatments with the two isotopes of Y-90 and Lu-177 linked either to DOTATOC or to DOTATATE administered in sequential treatment cycles or as a cocktail infusion for several cycles are feasible and may improve treatment outcomes, although they are also more toxic [137,138,139]. Several clinical studies are also exploring the combination of 177Lu-DOTATATE with chemotherapy (fluoropyrimidines alone or with temozolomide) or targeted agents such as everolimus [140,141,142]. PRRT is also being assessed, alone or in combination with chemotherapy, in the neoadjuvant setting with some encouraging results [143]. Promising results have been reported for the combination of CAPTEM with 177Lu-DOTATATE with ORRs reported in up to 80% of P-NETs [130]. Based on these encouraging results, the Australasian Gastrointestinal Trials Group (AGITG) designed the CONTROL NET Study, a Phase II randomized (2:1) exploratory study evaluating the activity of 177Lu-Octreotate and CAPTEM in two patient cohorts [144]. The P-NET cohort (*n* = 27) was randomized (2:1) to receive PRRT and CAPTEM vs. CAPTEM alone, and the midgut cohort (*n* = 45) was randomized (2:1) to receive PRRT and CAPTEM vs. PRRT alone. Recently presented preliminary results showed numerically higher ORR for the combination in both patient cohorts with no clear PFS benefit. Longer follow-up is needed to adequately assess whether the increased ORR is translated or not to a clinically meaningful PFS benefit to justify the increased toxicity observed with the combination.

Other SSTR radiolabeled agonists, such as 177Lu-Edotreotide or 177Lu-DOTATOC, have shown promising activity in NETs and are currently being assessed in GEP-NETs versus everolimus in the phase III COMPETE randomized trial (NCT03049189). Additionally, PRRT with alpha particle-emitting radionuclides (i.e., Bismuth-213 or Actinium-225) is also being actively developed as alpha particles are characterized by the emission of high-energy with a short-range, thereby allowing high-precision potent targeted therapy, avoiding the irradiation of normal surrounding tissues [145]. These isotopes have extremely high cytotoxic activity at the cellular level and may overcome resistance to PRRT using beta-emitting isotopes [146,147].

An alternative strategy to improve the absorption and binding of radiopharmaceuticals to NET cells include the intra-arterial (IA) administration of these agents in patients with liver-dominant disease. Several non-controlled studies have evaluated the administration of different radiopharmaceuticals through the hepatic artery (e.g., 177Lu-DOTATATE, 90Y-DOTATOC, etc.) with promising results [148,149,150,151,152]. Currently, the randomized LUTIA study is comparing the tumor-absorbed dose in liver metastases after intra-arterial administration of 177Lu-DOTATATE to that achieved after conventional intravenous administration [152]. The results of this trial are awaited with great interest and may potentially lead to the development of a large phase 3 trial to investigate the long-term outcome of IA PRRT.

In general, PRRT is considered a safe treatment option. However, some short- and long-term side effects have to be beared in mind and carefully considered. Bone marrow and renal toxicity are more commonly grade 1 and 2, but they may be transient or persistent [153]. The co-infusion of amino acids during the isotope infusion was demonstrated to reduce the risk of nephrotoxicity, although it was associated with manageable nausea and vomiting [154]. Notably, 5–21% of patients who had previously received chemotherapy developed grade 3 or 4 haematological toxicity [155]. Patients with bone metastases present a higher risk of myelotoxicity [156]. Finally, acute myeloid leukemia (AML) and myelodysplastic syndrome (MDS) are severe long-term complications related to PRRT and were reported to occur in approximately 0.5 and 1.5% of patients, respectively, after a median of 55 and 28 months following PRRT [11,136,157]. Patients that have received prior alkylating chemotherapy are at greater risk of developing MDS/acute leukemia following PRRT [158,159].

#### 4.3.2. Antagonists (177Lu-Satoreotide Tetraxetan, 177Lu-DOTA-LM3)

Radiolabeled SSTR2 antagonists, such as 177Lu-satoreotide tetraxetan, have shown higher tumor uptake, independent of SSTR activation, and greater tumor-to-organ ratios than agonists in preclinical models [160]. SSTR antagonists such as 68Ga-DOTA-JR11, [18F] AlF-NOTA-JR11, 68Ga-NODAGA-LM3 and 68Ga-DOTA-LM3, have demonstrated great sensitivity for the detection of NENs, potentially superior to [68Ga]Ga-DOTATATE [161,162,163,164]. A phase I study evaluated the efficacy and safety of 177Lu-satoreotide tetraxetan in 20 patients with advanced SSTR2-positive NETs [165]. Six patients received one cycle and 14 received two. The maximum activity per cycle was 7.4 GBq. However, grade 4 hematologic toxicity occurred in four of the seven (57%) patients after cycle 2. The study was suspended, and the protocol modified to limit the cumulative absorbed bone marrow dose to 1 Gy and to reduce prescribed activity for cycle 2 by 50%. The ORR was 45% (5% CR and 40% PR) and the median PFS was 21 months. Additional studies are ongoing to determine the optimal therapeutic dose and schedule [95]. Another study evaluated the safety and activity of the 177Lu-labeled somatostatin receptor (SSTR) antagonist DOTA-p-Cl-Phe-cyclo (D-Cys-Tyr-D-4-amino-Phe(carbamoyl)-Lys-Thr-Cys)D-Tyr-NH2 (177Lu-DOTA-LM3) [166]. Fifty-one patients with metastatic NENs received PRRT with 177Lu-DOTA-LM3, with good tolerance and promising activity.

## 5. Immunotherapy and Antibody-Drug Conjugates

### 5.1. Immune Check Point Inhibitors

Several immune check-point inhibitors (ICIs) have been demonstrated over the past decade to significantly improve the survival of patients with a wide spectrum of tumor types, including, among others, melanoma [167,168], renal and urothelial cancer [169], non–small cell lung cancer [170] and tumors with high microsatellite instability (MSI-h) or high tumor mutational burden (TMB-h) [171]. The immune response is tightly regulated by a fine balance between stimulating and inhibitory signals that also play a very relevant role in cancer surveillance and control. The Programmed Death-1 (PD-1) pathway is essential for maintaining peripheral T cell tolerance and is critical for attenuating autoimmunity and maintaining T cell homeostasis. However, this pathway also limits anti-tumor immunity. The PD-1 receptor is expressed on activated T cells and interacts with its ligand (PD-L1), expressed in tumor and immune cells, to down-regulate T-cell activation and promote tumor immune escape [172].

The immune landscape is highly heterogeneous in NENs. PDL-1 expression is low in G1-2 NET, whereas it is often high in G3 NENs [173,174]. Additionally, TMB is also higher in G3 NENs. Consistent with these observations, encouraging results have been reported with ICIs in different settings of high-grade NEN such as small cell lung cancer (SCLC) [175] or Merkel cell carcinoma (MCC) [176,177]. Indeed, several ICIs (i.e., atezolizumab or durvalumab) have been demonstrated to improve the survival of patients with extensive-stage SCLC when added to platinum-based chemotherapy and have been approved to treat SCLC in this context. In 2017, FDA approval was also granted for both the PD-L1 inhibitor avelumab and the PD-1 inhibitor pembrolizumab for the treatment of metastatic MCC [176]. Other PD-1 inhibitors such as nivolumab are also effective and are recommended for the treatment of advanced MCC in current National Comprehensive Cancer Network guidelines [177].

Many ICIs have also been tested in NENs (Table 4*)*. Results have been rather disappointing in G1-2 NETs [178,179,180] with the sole exception of spartalizumab (anti-PD1 antibody) in a small cohort of lung carcinoids that reported an ORR of 20% [181]. Single-agent treatment with ICIs has also been essentially ineffective in G3 NENs [182,183], except for toripalimab (anti-PD1 antibody), that reported an overall ORR in NENs with Ki-67 > 10% of 20% and greater for PDL1-positive tumors or tumors with high TMB (50 and 75%, respectively) [184].

More encouraging results have been reported for NEN patients treated in basket trials with dual CTLA4 and PD1 blockades (ipilimumab and nivolumab), with an ORR of 24–25%; responses were notably higher in G3 NENs and in those of lung origin [185]. However, the recently reported results from the DUNE basket trial (durvalumab and ipilimumab) conducted exclusively in GEP NENs have not confirmed these positive results [186]. Several other studies are currently testing novel treatment strategies with different combinations of ICIs, angiogenesis inhibitors or chemotherapy in GEP-NETs, lung carcinoids and extrapulmonary NECs (Table 4). The results of these trials shall help clarify the role, if any, of immunotherapy in NENs.

### 5.2. Antibody-Drug Conjugates Targeting SSTRs or DLL3

Antibody–drug conjugates (ADC) are a class of agents that consists of a mAb conjugated to a cytotoxic drug via a chemical linker. The monoclonal antibody directs the cytotoxic payload towards a target antigen expressed on the cancer cell surface, thereby reducing systemic drug exposure and therefore toxicity. This approach has shown to be effective in different types of cancers and is also being explored in NENs. Delta-like protein 3 (DLL3) is a Notch ligand that is expressed in tumor-initiating cells and > 80% of SCLC and other high grade NECs (lung, ovarian, prostate, bladder, etc.), with minimal to no expression in normal tissues [187,188,189]. Notch signaling regulates stem cell differentiation and self-renewal and is involved in cell–cell communication and cell-fate decisions during development. DLL3 is an atypical Notch receptor family ligand that, unlike related family members, inhibits Notch receptor activation. Delta-like protein 3 (DLL3)-targeted ADC rovalpituzumab tesirine (Rova-T) was initially tested in small cell lung cancer (SCLC) with some encouraging results, although randomized pivotal trials failed to demonstrate a survival benefit versus standard of care in pretreated patients and the development of this drug was halted [190,191]. At ESMO 2017, preliminary results of a phase I-II study of Rova-T were presented. This study planned to include several expansion cohorts of patients with different DLL3-positive high grade non-pulmonary NECs. However, this trial has not been published to date and no further data update has been presented since [192].

Another interesting target in NETs for ADC therapy is SSTRs. PEN-221 is a peptide–drug conjugate designed to target cancer cells via an SSTR2-targeting ligand conjugated to the antimicrotubular cytotoxic agent, DM1. In vitro, PEN-221 treatment of SSTR2-positive cells resulted in PEN-221 internalization and receptor-dependent inhibition of cellular proliferation. In vivo, PEN-221 exhibited rapid accumulation in SSTR2-positive SCLC xenograft tumors with quick clearance from plasma [193]. These data suggest potential for antitumor activity of PEN-221 in patients with SSTR2-positive tumors. With this rationale, a phase I/II study (NCT02936323) is currently exploring the activity of PEN-221 in SSTR2-expressing advanced cancers including NETs, pheochromocytomas and SCLC [194]. Other promising strategies at earlier stages of clinical development include bispecific antibodies or bites targeting CD3 and DLL3 or SSTR2 that will hopefully provide new therapeutic options for patients in the very near future. Other currently ongoing trials with novel targeted agents or novel combinations are summarized in Table 5.

## 6. Future Perspectives and Conclusions

Recent advances in understanding the biology of NENs have opened new avenues for the development of new therapeutic strategies that have substantially expanded the treatment armamentarium of these patients, including SSA, PRRT, mTOR and angiogenesis inhibitors. However, available treatment options are still rather limited, and all patients eventually develop resistance to these agents. Major efforts shall be made to overcome resistance and to develop innovative strategies to improve the treatment benefit–risk ratio in these patients, including the identification of novel targets for therapy and of biomarkers that allow an improved selection of patients for personalized patient care. The optimal sequence and/or treatment combinations are other pending issues, as are strategies to increase efficacy and minimize drug toxicity to improve patient outcomes, including quality of life.

## Figures and Tables

**Table 1:** Randomized phase II/III trials of mTOR inhibitors in neuroendocrine tumors (NETs).

Study	Design	Population	*n*	Drugs	ORR	*p* Value	PFS	HR	*p* Value	OS	HR	*p* Value
Pavel et al., 2011 RADIANT-2 [60]	Phase 3 randomized	Functioning NETs	420	Everolimus–Octreotide Placebo–Octreotide	3% 2%	NS	16.4 m 11.3 m	0.77	0.026	71% (18 m) 74% (18 m)	1.22	NS
Yao et al., 2011 RADIANT-3 [15]	Phase 3 randomized	Pancreatic NETs	410	Everolimus Placebo	5% 2%	NS	11.0 m 4.6 m	0.34	<0.001	NR	1.05	NS
Yao et al., 2016 RADIANT-4 [16]	Phase 3 randomized	Lung/Intestinal NETs (non-functioning)	302	Everolimus Placebo	2% 1%	NS	11.0 m 3.9 m	0.48	<0.001	NR NR	0.64	0.037
Kulke et al., 2019 COOPERATE-2 [62]	Phase 2 randomized	Pancreatic NETs	160	Everolimus–Pasireotide Everolimus	20% 6%	NA	16.8 m 16.6 m	0.99	NS	NR NR	0.93	NS
Ferolla et al., 2017 LUNA trial [63]	Phase 2 randomized	Lung or thymic NETs	124	Everolimus–Pasireotide Everolimus Pasireotide	2.4% 2.4% 2.4%	NA	11.8 m 12.5 m 8.5 m	NA	NA	NA	NA	NA
Salazar et al., 2018 NCT01628913 [64]	Phase 2 randomized	Pancreatic NETs	62	Dactolisib (BEZ235) Everolimus	9.7% 9.7%	NS	8.2 m 10.8 m	1.53	NA	96.6% (6 m) 90.3% (6 m)	NA	NS
Kulke et al., 2010 CALGB 80701 [65]	Phase 2 randomized	Pancreatic NETs	150	Everolimus–Bevacizumab Everolimus	31% 12%	0.005	16.7 m 14.0 m	0.80	0.12	36.7 m 35.0 m	0.72	NS

Abbreviations: PFS: progression free survival, OS: overall survival, ORR: overall response rate, CR: complete response, PR: partial response, SD: stable disease, LAR: long-acting repeatable, NS: not significant, NR: not reached, NA: not available.

**Table 2:** Randomized phase II-III trials of antiangiogenic agents in NETs.

Study	Design	Population	*n*	Drugs	ORR	*p*	PFS	HR	*p*	OS	HR	*p*
Raymond et al., 2011 SUN1111 [14]	Phase 3 randomized	Pancreatic NETs	171	Sunitinib Placebo	9.3% 0%	<0.007	11.4 m 5.5 m	0.42	<0.001	30.5 m 25.4 m	0.74	NS
Xu et al., 2020 SANET-P [90]	Phase 3 randomized	Pancreatic NETs	195	Surufatinib Placebo	NA	NA	NA	NA	NA	NA	NA	NA
Xu et al., 2020 SANET-EP [91]	Phase 3 randomized	Extra-pancreatic NET	198	Surufatinib Placebo	10.3% 0%	NA	9.2 m 3.8 m	0.33	<0.0001	NA	NA	NA
Garcia-Carbonero et al., 2020 AXINET [92]	Phase 2–3 randomized	Extra-pancreatic NET	256	Axitinib–Octreotide Placebo–Octreotide	17.5% 3.8%	0.0004	17.2 m 12.3 m	0.82	NS	NA	NA	NA
Bergsland et al., 2019 A021202 [93]	Phase 2 randomized	Extra-pancreatic NET	171	Pazopanib Placebo	2.1% 0%	NA	11.6 m 8.5 m	0.53	0.0005	41.3 m 42.4 m	1.13	NS
Yao et al., 2017 SWOG S0518 [94]	Phase 3 randomized	NETs (all sites)	427	Bevacizumab–Octreotide Interferon-α-2b–Octreotide	13% 4%	0.008	16.6 m 15.4 m	0.93	NS	35.2 m NR	1.16	NS
Kulke et al., 2015 CALGB 80701 [95]	Phase 2 randomized	Pancreatic NETs	150	Everolimus–Bevacizumab Everolimus	31% 12%	0.005	16.7 m 14.0 m	0.80	0.12	36.7 m 35.0 m	0.72	NS

Abbreviations: CR: complete response, LAR: long-acting repeatable, NA: not available, NR: not reached, NS: not significant, PFS: progression-free survival, ORR: overall response rate, OS: overall survival, PR: partial response, SD: stable disease.

**Table 3:** Randomized phase II-III trials of somatostatin receptors (SSTR)-targeted agents in NETs.

Study	Design	Population	*n*	Drug	ORR	*p*	PFS	HR	*p*	OS	HR	*p*
Rinke et al., 2009 PROMID study [9]	Phase 3 randomized	G1 midgut NETs	85	Octreotide LAR (30 mg/4 w) Placebo	2.4% 2.3%	NS	14.3 m 6.0 m	0.34	<0.001	84.7 m 83.0 m	0.81	NS
Caplin et al., 2014 CLARINET study [10]	Phase 3 randomized	GEP-NETs (Ki-67 < 10%)	204	Lanreotide ATG (120 mg/4 w) Placebo	1.9% 0%	NA	NR 18.0 m	0.47	0.001	NA	NA	NA
Wolin et al., 2015 NCT00690430 [129]	Phase 3 randomized *	Functioning NETs (refractory CS)	88	Pasireotide LAR (60 mg/4 w) Octreotide LAR (40/4 w)	2.0% 3.8%	NS	11.8 m 6.8 m	0.46	0.045	NA	NA	NA
Kulke et al., 2019 COOPERATE-2 trial [62]	Phase 2 randomized	Pancreatic NETs	160	Everolimus–Pasireotide Everolimus	20%6%	NA	16.8 m 16.6 m	0.99	NS	NRNR	0.93	NS
Ferolla et al., 2017 LUNA study [63]	Phase 2 randomized	Lung/thymic NETs	124	Everolimus–Pasireotide Everolimus Pasireotide	2.4% 2.4% 2.4%	NA	11.8 m 12.5 m 8.5 m	NA	NA	NA	NA	NA
Strosberg et al., 2017 NETTER-1 trial [13]	Phase 3 randomized	Midgut NETs	229	177Lu-Oxodotreotide Octreotide LAR (60/4 w)	18%3%	0.001	28.0 m 8.4 m	0.21	<0.001	NA	0.4	0.004
Pavlakis et al., 2020 CONTROL NET trial [130]	Phase 2 randomized	Pancreatic cohort Midgut cohort	27 45	PRRT–CAPTEM CAPTEM PRRT–CAPTEM PRRT	67% 33% 31% 15%	NS NS	76% (1y) 67% (1y) 92% (15 m) 90% (15m)	NANA	NS NS	NA	NA	NA

Abbreviations: CR: complete response, CS: carcinoid syndrome, LAR: long-acting repeatable, m: month, mg: milligrams, NA: not available, NR: not reached, NS: not significant, ORR: overall response rate, OS: overall survival, PFS: progression free survival, PR: partial response, SD: stable disease, w: week. * early termination at interim analysis for futility (primary endpoint: symptom control).

**Table 4:** Summary of relevant published trials with immune check-point inhibitors in advanced NETs and extrapulmonary neuroendocrine carcinomas (NECs).

Study	Drug	Population	*n*	Phase	Line	ORR	PFS (Median)	OS (Median)
KEYNOTE 028, 2019 [172]	Pembrolizumab	PDL1-positive EP-NETs PDL1-positive P-NETs	25 16	Ib	2 + line	12% 6.3%	5.6 m 4.5 m	21.1 m 21.0 m
KEYNOTE 158, 2020 [178]	Pembrolizumab	NETs (16% PDL1-positive)	107	II	Any line	3.7% 40% TMB-high (*n* = 5) 1.3% TMB-low (*n* = 80)	4.1 m	24.2 m
NCT02939651, 2020 [182]	Pembrolizumab	G3 NENs	29	II	2 + line	3.4%	2.2 m	5.1 m
CPDR001E2201, 2019 [181]	Spartalizumab (PDR001)	Lung-NETs P-NETs GI-NETs GEP NECs	30 33 32 21	II	2 + line	20% 3% 0% 5%	NA	NA
NCT03167853, 2020 [18]	Toripalimab (JS001)	NENs (Ki67 > 10%)	40	Ib	2 + line	43% (PD-L1 ≥ 10%) 8.3% (PD-L1 < 10%) 50% (G3 NENs) 75% (high TMB)	3.8 m (PD-L1 ≥ 10%) 2.2 m (PD-L1 < 10%)	9.1 m (PD-L1 ≥ 10%) 7.2 m (PD-L1 < 10%)
AVENEC, 2019 [183]	Avelumab	G3 NENs (except SCLC)	60	II	2nd line	6.9%	4 m	7 m
NCT03074513, 2020 [180]	Atezolizumab and bevacizumab	P-NETs EP-NETs	20 20	II	2 + line	20% 15%	19.6 m 14.9 m	NA
DART/SWOG 1609, 2020 [179]	Ipilimumab and nivolumab	NENs (all) G3 G1-2	32 18 14	II	Any line	25% 45% 0%	4 m	11 m
CA209-538, 2020 [96]	Ipilimumab and nivolumab	NENs (all) G3 lung NETs	29 13 9	II	Any line	24% 31% 33%	4.8 m	14.8 m
DUNE Trial 2020 [186]	Durvalumab and tremelimumab	Lung NETs GI NETs P-NETs GEP/unknown NECs	27 31 32 33	II	2 + line	0% 0% 6.9% 7.2%	5.3 m 8.0 m 8.1 m 2.5 m	NA

Abbreviations: EP-NETs: extrapancreatic NETs, GEP: gastroenteropancreatic, GI: gastrointestinal, LAR: long-acting repeatable, NA: not available, NET: neuroendocrine tumor, NEC: neuroendocrine carcinoma, NR: not reached, NS: not significant, ORR: objective response rate, OS: overall survival, P-NET: pancreatic NETs, PFS: progression free survival, SCLC: small cell lung cancer.

**Table 5:** Relevant ongoing trials with targeted therapy in advanced NENs.

Study	Population	Drug	Type/Phase	Line	Estimated *n*	Primary Endpoint
NETTER 2 trial NCT03972488	High G2/lowG3 GEP NETs (Ki-67 11–55%)	177Lu-DOTATATE vs. High dose Octreotide (60 mg every 28 days)	Randomized, phase III study	Any line	222	PFS
COMPETE trial NCT03049189	Non-functioning GI NETs, functioning or not P-NETs	177Lu-Edotreotide everolimus	Randomized, phase III study	Any line (progression required)	300	PFS
NCT04375267	SSTR + NETs	177Lu-DOTATATE plus olaparib	I	Any line	18	Safety
OCCLURANDOM trial NCT02230176	P-NETs	177Lu-DOTATATE vs. sunitinib	Randomized, phase II study	Any line (progression required, only one line of previous chemotherapy)	80	PFS
LU-CA-S trial NCT02736448	GEP-NETs	177Lu-DOTATATE plus capecitabine followed by SSA vs. 177Lu-DOTATATE followed by SSA	Randomized, phase II study	Any line	176	PFS
NCT04194125	GEP-NETs	177Lu-DOTATATE plus CAPTEM	Not randomized, phase II study	Any line	25	PFS
NCT03466216	NETs	AlphaMedix (²¹²Pb-DOTATATE)	I	Any line	50	Safety
CABINET trial NCT03375320)	Advanced NETs	Cabozantinib vs. placebo	Randomized, phase III	Any line (progression required)	395	PFS
NCT04427787	GEP-NETs	Cabozantinib plus lanreotide ATG	Not randomized, phase II study	Any line	69	ORR, Safety
NCT03891784	NETs	Abemaciclib	Not randomized, phase II study	Any line	37	ORR
NCT03950609	NETs	Lenvatinib plus everolimus	Not randomized, phase II study	Any line (progression required)	32	ORR
NCT03600233	NETs	CVM-1118	Not randomized, phase II study	Any line	30	PFS
HORMONET study NCT03870399	Hormone receptor positive NETs	Tamoxifen	Not randomized, phase II study	Any line	22	DCR at week 24
NCT03420521	NETs	Nivolumab and ipilimimab	II	Any line	64	ORR
NCT03591731	NECs	Nivolumab versus nivolumab and ipilimimab	II	Any line	180	ORR
NCT04207463	NETs	AK105 (antiPD-1) plus anlotinib (multi-TKI)	Not randomized, phase II study	Any line	150	ORR
NCT03475953	GEP-NETs (solid tumors)	Regorafenib with avelumab	Not randomized, phase I/II study	Any line	362 (solid tumors)	Safety
NCT03074513	Rare tumors, including NETs and NECs	Atezolizumab and bevacizumab	II	Any line	164	ORR
CABATEN trial, NCT04400474	NETs, NECs	Cabozantinib plus atezolizumab	Not randomized, phase II study	Any line	144	ORR
NCT04197310	NETs excluding P-NETs	Cabozantinib plus nivolumab	Not randomized, phase II study	Any line	35	ORR
NCT04079712	NECs	Cabozantinib plus nivolumab plus ipilimumab	Not randomized, phase II study	Any line	30	ORR
NCT04197310	Extra-pancreatic NETs	Nivolumab and cabozantinib	II	Any line	35	ORR
NCT03290079	Lung and GI NETs	Pembrolizumab and lenvatinib	II	Any line	35	ORR
NCT03879057	Advanced solid tumors including NENs	Toripalimab and surufatinib	I	Any line	24	Safety
NCT03910660	Prostate NECs	Talabostat mesylate plus pembrolizumab	Not randomized, I/II study	Any line	40	ORR
NCT03411915	NETs	Tidutamab (XmAb18087) (anti-SSTR2 x anti-CD3 monoclonal antibody)	I	Any line	87	Safety
NCT03879694	Lung and P-NETs	Octreotide LAR plus sargramostim plus SVN53-67/M57-KLH Peptide Vaccine	I	Any line	10	Safety
NCT03992911	Bladder NECs	Ph.II simmtecan, 5-FU and l-LV plus toripalimab Ph.III simmtecan, 5-FU and l-LV plus toripalimab Ph.III etoposide versus etoposide, cisplatin	Not randomized, phase II study; Randomized phase III study	Any line	336	OS
NCT03582475	Prostate/bladder NECs	Chemotherapy (platinum–etoposide or docetaxel) plus pembrolizumab	Not randomized, phase I study	Any line	30	ORR, PFS, OS
NICE-NEC GETNE T1913	NECs	carboplatinum–etoposide plus nivolumab	Not randomized, phase II study	I	38	ORR
NCT03901378	NECs (excluding SCLC)	Pembrolizumab and platinum–etoposide	II	I	36	PFS
NCT03728361	NECs	Temozolomide plus nivolumab	Not randomized, phase II study	Any line	53	ORR

Abbreviations: DCR: disease control rate, GEP: gastroenteropancreatic, GI: gastrointestinal, LAR: long-acting repeatable, NET: neuroendocrine tumor, NEC: neuroendocrine carcinoma, ORR: objective response rate, OS: overall survival, P-NET: pancreatic NETs, PFS: progression free survival, SSTR2: somatostatin receptor 2.
